# COVID-19 critical illness in pregnancy

**DOI:** 10.1177/1753495X211051246

**Published:** 2021-11-02

**Authors:** Stephen E Lapinsky, Maha Al Mandhari

**Affiliations:** 1 Faculty of Medicine, 12366 University of Toronto, Canada; 2 Intensive Care Unit, Mount Sinai Hospital, Canada; 3 Interdepartmental Division of Critical Care Medicine, University of Toronto, Canada

**Keywords:** Pregnancy complications, infectious, COVID-19, respiratory failure, pandemic, critical care

## Abstract

Although the pregnant population was affected by early waves of the COVID-19 pandemic, increasing transmission and severity due to new viral variants has resulted in an increased incidence of severe illness during pregnancy in many regions. Critical illness and respiratory failure are relatively uncommon occurrences during pregnancy, and there are limited high-quality data to direct management. This paper reviews the current literature on COVID-19 management as it relates to pregnancy, and provides an overview of critical care support in these patients. COVID-19 drug therapy is similar to that used in the non-pregnant patient, including anti-inflammatory therapy with steroids and IL-6 inhibitors, although safety data are limited for antiviral drugs such as remdesivir and monoclonal antibodies. As both pregnancy and COVID-19 are thrombogenic, thromboprophylaxis is essential. Endotracheal intubation is a higher risk during pregnancy, but mechanical ventilation should follow usual principles. ICU management should be directed at optimizing maternal well-being, which in turn will benefit the fetus.

Many regions are experiencing an increase in critical illness related to COVID-19 during pregnancy.^1–3^ Literature on the management of COVID-19 related critical illness in pregnancy is relatively limited. This narrative review examines the available literature to provide practical information for the healthcare practitioner managing severe COVID-19 infection during pregnancy. We aim to provide pregnancy-specific information for those familiar with managing COVID-19 and COVID-19-specific knowledge for the obstetrician or maternal medicine specialist.

## COVID-19 in the pregnant patient

Initial data relating to SARS-CoV2 infection in the first wave of the pandemic did not appear to demonstrate a marked increase in the risk of severe disease or mortality in the pregnant population. However, knowledge is constantly evolving and more recent data suggest an increased risk of hospitalization, ICU admission, and respiratory failure.^1,3,4^ A meta-analysis of 42 studies demonstrates that COVID-19 produces a higher risk of maternal and perinatal morbidity, including preeclampsia, preterm birth, stillbirth, low birth weight infants, and neonates requiring NICU admission.^4^ Data from 499 U.S. academic medical centers over the first year of the pandemic identified an increased risk in pregnant women with COVID-19 (compared with pregnant women without the infection) of ICU admission (odds ratio 5.84), mechanical ventilation (odds ratio 14.3), and in-hospital mortality (odds ratio 15.4).^5^ About 10–20% of infected pregnant women develop moderate to severe disease requiring a period of hospitalization. Most of those who developed the significant disease (in the initial waves of infection) have had associated medical comorbidities, most commonly obesity, diabetes, chronic hypertension, or immune suppression.^1,3^ A recent retrospective review of 2020 data identified that adverse maternal outcomes were higher in high-risk pregnancies than in low-risk pregnancies. Pregnancies considered high-risk included conditions such as pre-existing diabetes mellitus, chronic hypertension, autoimmune diseases, and obstetric disorders such as preeclampsia, gestational hypertension, and gestational diabetes mellitus.^6^

The evolving picture of disease severity may be related to an increased prevalence of mutations (“variants of concern”—VOC) which appear to be associated with increased transmission of the virus and increased severity of the disease.^7^ In areas/periods where VOC have become prevalent, an increased incidence of severe disease and ICU admission has been noted amongst pregnant individuals.^8^

## Physiology and pathophysiology

Some physiological effects of pregnancy, such as nasal congestion and physiological dyspnea may mimic some of the clinical features of COVID-19. Anatomic changes occur in pregnancy which may affect the management of the patient with COVID-19. The upper airway becomes edematous and friable in pregnancy making intubation more difficult,^9^ and these effects may be exacerbated by preeclampsia and during labor.

The pregnant woman has increased minute ventilation mediated by an increased tidal volume. Respiratory rate is not increased by pregnancy, and the median rate is 15 breaths/min with the 97th percentile at 22/min.^10^ Tachypnea should therefore not be attributed to the pregnant state. However, it should be borne in mind that the majority (75%) of pregnant women develop some degree of dyspnea by the third trimester.^11^ This dyspnea occurs as an isolated symptom, not associated with cough or abnormal findings on physical examination. The physiological increase in alveolar ventilation produces a respiratory alkalosis, with a PaCO_2_ at around 30 mmHg (4 kPa). This hypocapnia facilitates a gradient to allow for placental excretion of fetal CO_2._^9^

Oxygenation is not adversely affected by pregnancy itself, but COVID-19 commonly causes severe hypoxemia due to an acute respiratory distress syndrome (ARDS)-like a picture. ARDS is characterized by an oxygenation deficit of acute onset, with bilateral radiographic infiltrates that are not due to a cardiac cause. The pathophysiology of ARDS in COVID may be somewhat different from conventional ARDS. An initially preserved lung compliance has been suggested, with ventilation-perfusion mismatch possibly exacerbated by microangiopathy and microthromboses, as well as by loss of the normal protective hypoxic vasoconstrictor response.^12,13^ Oxygen delivery to the placenta and fetus is determined by both maternal oxygen saturation and uterine blood flow, and the fetoplacental system and increased oxygen-carrying capacity of fetal hemoglobin can compensate to some degree for maternal hypoxemia. Although chronic hypoxemia has an adverse effect on the fetus,^14^ there are little data on the adverse effects of short-term episodes of oxygen desaturation. The effects on the fetus of maternal hypoxemia will be compounded in the presence of a reduced maternal cardiac output and blood flow to the placenta.

Pregnancy is a prothrombotic state, as is COVID-19. COVID-19 has been described as a thromboinflammatory condition, with loss of the normal antithrombotic and anti-inflammatory functions of endothelial cells, which leads to dysregulation of coagulation.^15^ The clinician should have an increased awareness of the risk of thrombosis. A high level of suspicion for thromboembolic complications may necessitate ultrasound or CT scan imaging, and adequate prophylaxis should be provided.

An increased incidence of preeclampsia has been described in women with COVID, but some features of COVID-19 infection may mimic preeclampsia.^3^ Elevated liver enzymes, thrombocytopenia, and a prolonged aPTT can be seen with both diseases. Blood pressure measurements, urine protein–creatinine ratio, and placenta growth factor (PlGF) can be used to identify preeclampsia.^16^ A decrease in PlGF levels is characteristic of preeclampsia, and elevated levels of PlGF have been documented in non-pregnant individuals with severe COVID-19.^17^

## Management of critical illness (Figure 1)

An overarching concept in the management of critically ill pregnant women is that optimizing the maternal status is beneficial for the fetus. Interventions should generally not be performed purely for fetal benefit, and essential management should not be altered due to concerns for perceived fetal harm. Close communication with Obstetricians and Maternal-Fetal Medicine specialists may help allay any concerns. Radiological investigations, including chest CT scans, should not be withheld during pregnancy if clinically valuable.^18^

**Figure 1.**
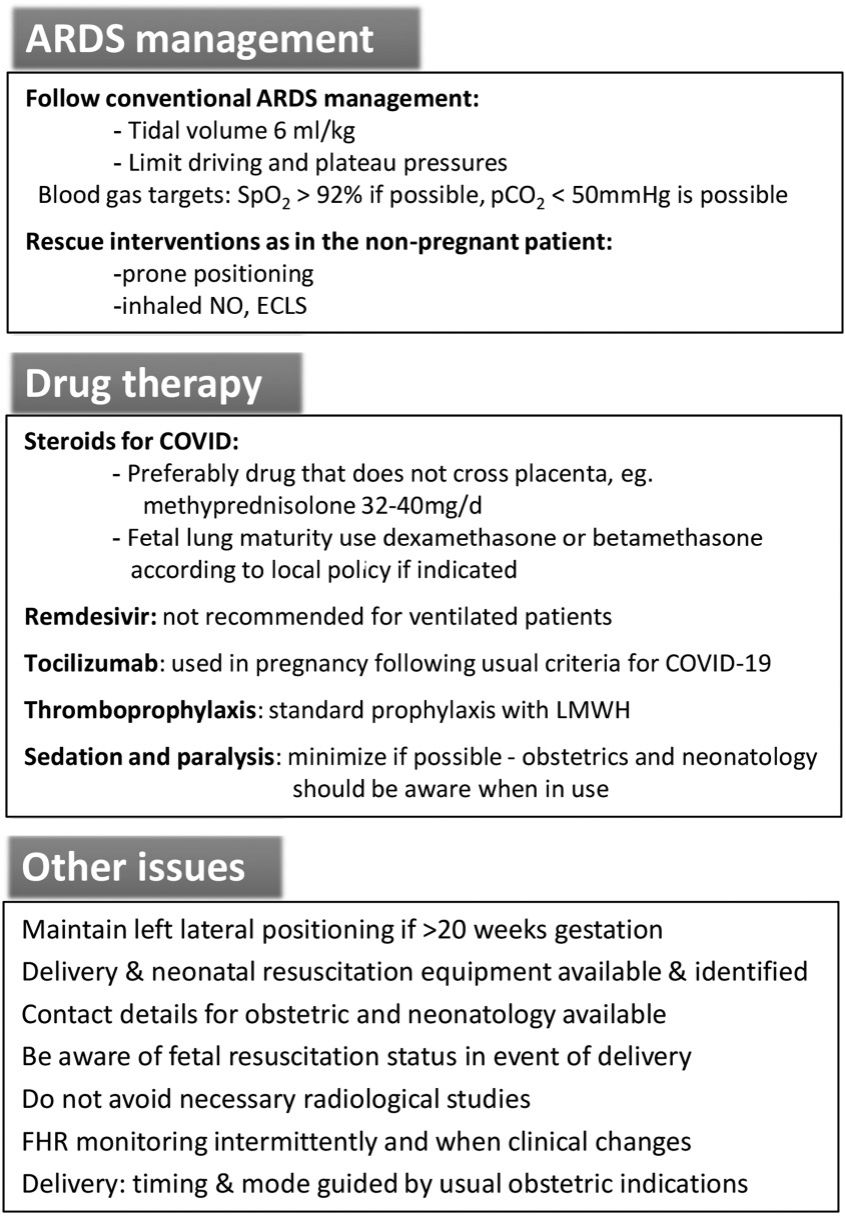
Principles of management for the pregnant patient with severe COVID-19 pneumonitis requiring mechanical ventilation.

ARDS: acute respiratory distress syndrome; SpO_2_: oxygen saturation; pCO_2_: partial pressure of carbon dioxide; NO: nitric oxide; ECLS: extracorporeal life-support; LMWH: low molecular weight heparin; FHR: fetal heart rate.

### Pharmacological management

The drug therapies currently available for the management of COVID-19 have not been studied in the pregnant population, and the suggestions below are based on the best available, but limited data.

#### Dexamethasone

An open-label randomized trial of dexamethasone (6 mg daily) in COVID-19 demonstrated reduced mortality in patients requiring oxygen or mechanically ventilation.^19^ Pregnant women were eligible for this trial, but the total numbers were very small. Steroid therapy for severe COVID-19 pneumonitis is appropriate in the pregnant population following these indications. Dexamethasone crosses the placenta, and for treatment of COVID-19 pneumonitis a drug that does not affect the fetus is preferable, for example, methylprednisolone 32–40 mg/day (equivalent to prednisolone 40–50 mg/day) to complete a ten-day steroid course.^20^ Depending on the gestational age, steroids (dexamethasone or betamethasone) may be used initially to promote fetal lung maturation, but this decision is best guided by the Obstetric team.

#### Remdesivir

In a randomized controlled trial that excluded pregnant subjects, remdesivir was shown to shorten the time to recovery in hospitalized patients requiring oxygen therapy.^21^ However, there is no consensus for its use in treatment guidelines, both in the non-pregnant and the pregnant patient. Pregnant subjects were excluded from all five randomized trials evaluating remdesivir for COVID-19 and safety data are limited, but use in pregnancy is not absolutely contraindicated.^22^ In a review of 67 women who received remdesivir during pregnancy (median gestation 28 weeks) under a compassionate release program in early 2020, no neonatal deaths or congenital abnormalities were identified.^23^ Treatment guidelines vary from discouraging the use of remdesivir in pregnancy to following a similar approach to the non-pregnant population, that is, to be used for moderate COVID-19 but not for severe disease or for patients on mechanical ventilation.

#### Tocilizumab

A randomized open-label trial of tocilizumab demonstrated improved survival in hospitalized COVID-19 patients with hypoxia and systemic inflammation.^24^ There are some pregnancy data available from the use of this IL-6 inhibitor in rheumatological conditions, suggesting that it is safe in pregnancy.^25^ There is a theoretical risk of neonatal immunosuppression and an increase in preterm delivery, but there are no reports of fetal malformations.^26^ Although pregnant women were not included in the clinical trials of tocilizumab in COVID-19, most guidelines recommend its use in severe COVID-19, based on rheumatological safety data.

#### Monoclonal antibodies

Monoclonal antibodies that target the spike protein have a clinical benefit in treating COVID-19, and several (casirivimab plus imdevimab, bamlanivimab plus etesevimab, and sotrovimab) are approved for use (or approved with certain restrictions) in many jurisdictions. Pregnancy-specific data does not exist, but as immunoglobulin (Ig) G antibodies, these drugs will be expected to cross the placenta after the expression of the neonatal Fc receptor from about mid-gestation. Some guidelines do recommend their use in pregnancy.^27^

#### Thromboprophylaxis

Both COVID-19 infection and pregnancy are prothrombotic states and the risk of coagulopathy and thromboembolism is increased.^28^ Thromboprophylaxis is strongly recommended in pregnant women with COVID-19, following usual practices with low molecular weight heparin. Data in the non-pregnant patient suggests the use of full anticoagulation in hospitalized (but not critically ill) patients, but only prophylactic treatment in the critically ill.^29,30^

#### Glycemic control

Pre-existing and gestational diabetes are considered risk factors for increased severity of COVID-19 during pregnancy and are associated with adverse outcomes. Pregnancy also produces altered maternal glucose homeostasis. This physiological change, combined with the stress response of acute illness and the use of corticosteroid therapy, results in hyperglycemia being very common in hospitalized patients with COVID-19. Glucose levels should be closely monitored and treated with insulin when required.

#### Vasopressor therapy

Inotropes and vasopressors will potentially reduce placental perfusion but may be essential to maintain end-organ perfusion (including the placenta). If vasopressor support is required, this therapy should not be withheld because of concerns for potential adverse effects on the fetus.

### Respiratory management

#### Oxygen therapy

Oxygen therapy in pregnancy is by the usual modalities, including non-rebreather masks and high-flow nasal cannulae (HFNC). Pregnant women may have significant nasal congestion, but HFNC still appears effective.^31^ Little data exist to identify appropriate oxygen targets (see below), and many references suggest a higher oxygen level than in the non-pregnant population. There is little evidence to support this, but there is evidence that hyperoxygenation may have adverse maternal hemodynamic effects in pregnancy.^32^

#### Non-invasive respiratory support

This includes face-mask and helmet continuous positive airway pressure (CPAP) and non-invasive bi-level ventilation, and has been used extensively for COVID-19 in the non-pregnant population in some regions, potentially avoiding intubation. A systematic review suggests that this is a feasible strategy.^33^ Literature on non-invasive respiratory support in pregnancy outside of COVID-19 is limited predominantly to case reports, but it is considered safe if the patient is alert and protecting their airway. The risk of aspiration always needs to be considered, but there is a potential benefit in avoiding intubation and sedation. Non-invasive respiratory support has been used effectively in pregnant women with COVID-19.^34^

#### Intubation and ventilation

It is well established that airway management in pregnancy may be challenging. A careful assessment of the airway and an early discussion of the intubation plan must be made with the most experienced operator, preferably an obstetric anesthesiologist if available. Concerns related to airway management in pregnancy include an edematous, friable airway, a higher risk of aspiration, and aortocaval compression with hemodynamic instability necessitating left uterine displacement.^35^ There is an increased risk of rapid oxygen desaturation due to a reduction in functional residual capacity (FRC) and increased oxygen consumption. This effect may be exacerbated in the hypoxic patient with COVID-19. There are no recommended changes to rapid sequence induction (RSI) dosing, but care must be taken to ensure adequate weight-based dosing of neuromuscular blockers to facilitate rapid intubation. The use of video laryngoscopy has been recommended in pregnant patients.^36^

Principles of mechanical ventilation are similar to the non-pregnant population, with a target tidal volume of 6 ml/kg based on ideal body weight. There is no evidence to suggest that a change in the mode of ventilation or altering monitoring parameters is necessary. It is possible that the pregnant patient may require a higher PEEP level to attain alveolar recruitment, and plateau pressure may be a little higher due to reduced respiratory system compliance.^37^ Prone positioning is feasible and effective during pregnancy.^38^ As in the non-pregnant patient, prone positioning is indicated for patients with severe hypoxemia, for example, partial pressure of arterial oxygen to the fraction of inspired oxygen ratio (PaO_2_/FiO_2_ ratio) of less than 150 mmHg. Inhaled bronchodilators (e.g. inhaled nitric oxide) can be used in pregnancy. Extracorporeal membrane oxygenation (ECMO) is an option during pregnancy and good maternal and fetal outcomes have been reported.^39^

#### Blood gas targets

Attaining a high oxygen target is often limited by maternal pathophysiology, and maternal oxygen saturation is only one component of oxygen delivery to the fetus. Aiming for an oxygen saturation greater than 95% in pregnancy is not evidence-based. Interventions to improve maternal oxygen saturation (e.g. high PEEP levels) may reduce cardiac output and placental perfusion, therefore ultimately not benefitting the fetus. The fetus can mitigate the effects of hypoxemia (even up to 50% reduction in oxygen content) by redirecting cardiac output to the fetal heart and brain.^40^ A further drop in oxygen content produces anaerobic metabolism, and if oxygen delivery is reduced by more than 75%, central nervous system damage may occur.

Permissive hypercapnia is an accepted approach to ventilating patients with ARDS to limit injurious tidal volumes,^41^ but concern exists in the pregnant patient as the normal PaCO_2_ level is reduced. There are limited data on the effects of hypercapnia in pregnancy, which can produce fetal respiratory acidosis. Although this acidosis may not have the same poor fetal implications as fetal lactic acidosis produced by hypoxemia, it can lead to a right-shift in the fetal hemoglobin oxygen dissociation curve, reducing the beneficial oxygen-carrying characteristics of fetal hemoglobin. Older clinical studies have demonstrated the lack of adverse fetal effects from mild hypercapnia (40–55 mmHg)^42,43^ and a case series documents successful pregnancy outcomes after significant short-term hypercapnia (PaCO_2_ 43–114 mmHg; 5.7–15.2 kPa).^44^ Managing hypercapnia often becomes a risk–benefit balance between injurious high tidal volumes and the potential adverse effects of hypercapnia; in practice we often allow PaCO_2_ to rise to 50 mmHg (6.7 kPa) in these patients. Hyperventilation with low PaCO_2_ levels reduces uterine blood flow and compromises fetal oxygenation, by alkalosis-induced uterine vasoconstriction and also by reduced cardiac output due to elevated intrathoracic pressure.^45^

Electronic fetal heart rate (FHR) monitoring (at an appropriate gestational age) may help evaluate the effects of abnormal blood gases on the fetus.

### Delivery

A multidisciplinary plan regarding possible obstetric delivery in the ICU should be made on admission. Close fetal monitoring by the obstetric service is essential. Severe COVID-19 infection alone is not an indication for urgent delivery and delivery may not improve maternal respiratory function.^37^ The decision to deliver should be based on usual maternal or fetal considerations. The following are issues to consider in planning for obstetric delivery in the ICU:

- Early discussion regarding the decision for fetal resuscitation should be made with the family (in conjunction with obstetrics and neonatology), and ICU staff should be aware of the neonatal resuscitation status which may change with advancing gestational age.
- Drugs and equipment for vaginal delivery, cesarean delivery, and neonatal resuscitation should be available in the ICU at all times, in the presence of a viable fetus.
- The decision to deliver and the mode of delivery should follow usual obstetric principles.
- Drug therapy used during labor and delivery may require reassessment in the presence of severe pneumonitis. Magnesium sulfate may exacerbate respiratory failure by causing muscle weakness at toxic levels. Carboprost (Hemabate®) may increase pulmonary vascular resistance and worsen the V/Q mismatch.
- Post-delivery autotransfusion may lead to fluid overload, increasing pulmonary edema, and worsening right ventricular function in the presence of ARDS-induced pulmonary hypertension.

## Conclusion

The management of COVID-19 during pregnancy requires collaborative multidisciplinary planning including critical care, obstetric medicine, maternal-fetal medicine, neonatology, and ethics involvement. Early communication with the patient and family is strongly advised. All efforts should aim at optimizing maternal care, which will, in turn, benefit the fetus. Maintaining the current principles of ARDS management is critical, with some modifications made based on pregnancy considerations.

## References

[1] Money D . Canadian Surveillance of COVID-19 in pregnancy: epidemiology, maternal and infant outcomes. Report #2. Public Health Agency of Canada, January 15, 2021. Available at: http://med-fom-ridprogram.sites.olt.ubc.ca/files/2021/01/CANCOVID_Preg-report-2-ON-AB-BC-QC-data_15JAN2021_FINAL.pdf (accessed 23 April 2021)

[2] Blitz MJ Grünebaum A Tekbali A , et al. Intensive care unit admissions for pregnant and nonpregnant women with coronavirus disease 2019. Am J Obstet Gynecol 2020; 223: 290–291. 32387323 10.1016/j.ajog.2020.05.004 PMC7204719

[3] Allotey J Stallings E Bonet M , et al. Clinical manifestations, risk factors, and maternal and perinatal outcomes of coronavirus disease 2019 in pregnancy: living systematic review and meta-analysis. BMJ 2020; 370: m3320. 32873575 10.1136/bmj.m3320 PMC7459193

[4] Wei SQ Bilodeau-Bertrand M Liu S Auger N . The impact of COVID-19 on pregnancy outcomes: a systematic review and meta-analysis. CMAJ 2021; 193(16): E540–E548. 33741725 10.1503/cmaj.202604 PMC8084555

[5] Chinn J Sedighim S Kirby KA , et al. Characteristics and outcomes of women with COVID-19 giving birth at US academic centers during the COVID-19 pandemic. JAMA Netw Open 2021; 4(8): e2120456. 34379123 10.1001/jamanetworkopen.2021.20456 PMC8358731

[6] D‘Antonio F Sen C Mascio DD , et al. Maternal and perinatal outcomes in high compared to low risk pregnancies complicated by severe acute respiratory syndrome coronavirus 2 infection (phase 2): the World Association of Perinatal Medicine working group on coronavirus disease 2019. Am J Obstet Gynecol MFM 2021; 3(4): 100329. 33621713 10.1016/j.ajogmf.2021.100329 PMC7896113

[7] Center for Disease Control and Prevention. SARS-CoV-2 Variant Classifications and Definitions. https://www.cdc.gov/coronavirus/2019-ncov/cases-updates/variant-surveillance/variant-info.html (April 2021, accessed 23 April 2021)

[8] Knight M Ramakrishnan R Bunch K , et al. Females in Hospital with SARS-CoV-2 infection, the association with pregnancy and pregnancy outcomes: A UKOSS/ISARIC/CO-CIN investigation. UK Scientific Advisory Group for Emergencies (SAGE). https://assets.publishing.service.gov.uk/government/uploads/system/uploads/attachment_data/file/977287/s1171-ukoss-isaric-co-cin-covid-19-young-females-pregnancy-report.pdf (April 2021, accessed 23 April 2021)

[9] Hegwald MJ Crapo RO . Respiratory physiology in pregnancy. Clin Chest Med 2011; 32: 1. 21277444 10.1016/j.ccm.2010.11.001

[10] Green LJ Mackillop LH Salvi D , et al. Gestation-specific vital sign reference ranges in pregnancy. Obstet Gynecol 2020; 135: 653–664. 32028507 10.1097/AOG.0000000000003721

[11] Milne JA Howie AD Pack AI . Dyspnoea during normal pregnancy. Br J Obstet Gynaecol 1978; 85: 260–263. 638094 10.1111/j.1471-0528.1978.tb10497.x

[12] Ackermann M Verleden SE Kuehnel M , et al. Pulmonary vascular endothelialitis, thrombosis, and angiogenesis in COVID-19. N Engl J Med 2020; 383: 120–128. 32437596 10.1056/NEJMoa2015432 PMC7412750

[13] Chiumello D Camporota L Gattinoni L Marini JJ . Complexity and unanswered questions in the pathophysiology of COVID-19 ARDS. Intensive Care Med 2021; 47: 495–496. 33527153 10.1007/s00134-021-06353-x PMC7849962

[14] Presbitero P Somerville J Stone S Aruta E Spiegelhalter D Rabajoli F . Pregnancy in cyanotic congenital heart disease. Outcome of mother and fetus. Circulation 1994; 89: 2673–6. 8205680 10.1161/01.cir.89.6.2673

[15] Connors JM Levy JH . Thromboinflammation and the hypercoagulability of COVID-19. J Thromb Haemost 2020; 18: 1559–1561. 32302453 10.1111/jth.14849 PMC9770920

[16] Kornacki J Wender-Ożegowska E . Utility of biochemical tests in prediction, diagnostics and clinical management of preeclampsia: a review. Arch Med Sci 2020 Aug 3; 16(6): 1370–1375. 33224336 10.5114/aoms.2020.97762 PMC7667413

[17] Smadja DM Philippe A Bory O , et al. Placental growth factor level in plasma predicts COVID-19 severity and in-hospital mortality. J Thromb Haemost 2021; 19(7): 1823–1830. 33830623 10.1111/jth.15339 PMC8250221

[18] Lowe S . Diagnostic imaging in pregnancy: Making informed decisions. Obstet Med 2019; 12(3): 116–122. 31523267 10.1177/1753495X19838658 PMC6734637

[19] Recovery Collaborative Group, Horby P Lim WS , et al. Dexamethasone in hospitalized patients with COVID-19. N Engl J Med 2021; 384: 693–704. 32678530 10.1056/NEJMoa2021436 PMC7383595

[20] D‘Souza R Ashraf R Rowe H , et al. Pregnancy and COVID-19: pharmacologic considerations. Ultrasound Obstet Gynecol 2021; 57: 195–203. 32959455 10.1002/uog.23116 PMC7537532

[21] Beigel JH Tomashek KM Dodd LE Mehta AK Zingman BS Kalil AC , et al. Remdesivir for the treatment of COVID-19 – Final Report. N Engl J Med 2020; 383: 1813–26. 32445440 10.1056/NEJMoa2007764 PMC7262788

[22] Jorgensen SCJ Davis MR Lapinsky SE . A review of remdesivir for COVID-19 in pregnancy and lactation. J Antimicrob Chemother 2021 Aug 24: dkab311. Online ahead of print. 34427297 10.1093/jac/dkab311 PMC8499800

[23] Burwick RM Yawetz S Stephenson KE Collier AY Sen P Blackburn BG , et al. Compassionate use of remdesivir in pregnant women with severe COVID-19. Clin Infect Dis 2020: ciaa1466. Epub ahead of print. 10.1093/cid/ciaa1466 PMC7797739 33031500

[24] Recovery Collaborative Group. Tocilizumab in patients admitted to hospital with COVID-19 (RECOVERY): a randomised, controlled, open-label, platform trial. Lancet 2021; 397(10285): 1637–1645. 33933206 10.1016/S0140-6736(21)00676-0 PMC8084355

[25] Förger F Villiger PM . Treatment of rheumatoid arthritis during pregnancy: present and future. Expert Rev Clin Immunol 2016; 12: 937–44. 27170517 10.1080/1744666X.2016.1184973

[26] Hoeltzenbein M Beck E Rajwanshi R , et al. Tocilizumab use in pregnancy: Analysis of a global safety database including data from clinical trials and post-marketing data. Semin Arthritis Rheum 2016; 46: 238–245. 27346577 10.1016/j.semarthrit.2016.05.004

[27] COVID-19 Treatment Guidelines: Anti-SARS-CoV-2 Monoclonal Antibodies, National Institutes of Health. https://www.covid19treatmentguidelines.nih.gov/therapies/anti-sars-cov-2-antibody-products/anti-sars-cov-2-monoclonal-antibodies (accessed 20 August 2021)

[28] Servante J Swallow G Thornton JG , et al. Haemostatic and thrombo-embolic complications in pregnant women with COVID-19: a systematic review and critical analysis. BMC Pregnancy Childbirth 2021; 21: 108. 33546624 10.1186/s12884-021-03568-0 PMC7863033

[29] The REMAP-CAP, ACTIV-4a, and ATTACC Investigators. Therapeutic anticoagulation with heparin in critically ill patients with COVID-19. N Engl J Med 2021; 385: 777–789. 34351722 10.1056/NEJMoa2103417 PMC8362592

[30] The ATTACC, ACTIV-4a, and REMAP-CAP Investigators. Therapeutic anticoagulation with heparin in noncritically ill patients with COVID-19. N Engl J Med 2021; 385: 790–802. 34351721 10.1056/NEJMoa2105911 PMC8362594

[31] Zhou S Zhou Y Cao X Ni X Du W Xu Z Liu Z . The efficacy of high flow nasal oxygenation for maintaining maternal oxygenation during rapid sequence induction in pregnancy: A prospective randomised clinical trial. Eur J Anaesthesiol 2020 Nov 24. Epub ahead of print 10.1097/EJA.0000000000001395 33259452

[32] McHugh A El-Khuffash A Bussmann N Doherty A Franklin O Breathnach F . Hyperoxygenation in pregnancy exerts a more profound effect on cardiovascular hemodynamics than is observed in the nonpregnant state. Am J Obstet Gynecol 2019; 220(4): 397.e1–397.e8. 10.1016/j.ajog.2019.02.059 30849354

[33] Cammarota G Esposito T Azzolina D , et al. Noninvasive respiratory support outside the intensive care unit for acute respiratory failure related to coronavirus-19 disease: a systematic review and meta-analysis. Crit Care 2021; 25(1): 268. 34330320 10.1186/s13054-021-03697-0 PMC8324455

[34] Keita H James A Bouvet L , et al. Clinical, obstetrical and anaesthesia outcomes in pregnant women during the first COVID-19 surge in France: a prospective multicentre observational cohort study. Anaesth Crit Care Pain Med 2021: 100937. Online ahead of print. 34391984 10.1016/j.accpm.2021.100937 PMC8359490

[35] Rajagopalan S Suresh M Clark SL , et al. Airway management for cesarean delivery performed under general anesthesia. Int J Obstet Anesth 2017; 29: 64. 27884665 10.1016/j.ijoa.2016.10.007

[36] Toker MK Altıparmak B Karabay AG . Comparison of the McGrath video laryngoscope and Macintosh direct laryngoscope in obstetric patients: A randomized controlled trial. Pak J Med Sci 2019; 35: 342. 31086512 10.12669/pjms.35.2.646 PMC6500838

[37] Lapinsky SE Rojas-Suarez JA Crozier TM , et al. Mechanical ventilation in critically-ill pregnant women: a case series. Int J Obstet Anesth 2015; 24: 323–8. 26355021 10.1016/j.ijoa.2015.06.009

[38] Tolcher MC McKinney JR Eppes CS , et al. Prone positioning for pregnant women with hypoxemia due to coronavirus disease 2019 (COVID-19). Obstet Gynecol 2020; 136: 259–261. 32516274 10.1097/AOG.0000000000004012

[39] Lankford AS Chow JH Jackson AM , et al. Clinical outcomes of pregnant and postpartum extracorporeal membrane oxygenation patients. Anesth Analg 2021; 132: 777–787. 33591093 10.1213/ANE.0000000000005266

[40] Peeters LL Sheldon RE Jones MD Jr Makowski EL Meschia G . Blood flow to fetal organs as a function of arterial oxygen content. Am J Obstet Gynecol 1979; 135: 637–646. 507116 10.1016/s0002-9378(16)32989-1

[41] Hickling KG . Permissive hypercapnia. Respir Care Clin N Am 2002; 8: 155–69. 12481813 10.1016/s1078-5337(02)00006-0

[42] Peng AT Blancato LS Motoyama EK . Effect of maternal hypocapnia v. eucapnia on the foetus during caesarean section. Br J Anaesth 1972; 44: 1173–8. 4647112 10.1093/bja/44.11.1173

[43] Ivankovic AD Elam JO Huffman J . Effect of maternal hypercarbia on the newborn infant. Am J Obstet Gynecol 1970; 107: 939–46. 5429022 10.1016/s0002-9378(16)34052-2

[44] Elsayegh D Shapiro JM . Management of the obstetric patient with status asthmaticus. J Intensive Care Med 2008; 23(6): 396–402. 18794165 10.1177/0885066608324295

[45] Levinson G Shnider SM DeLorimier AA Steffenson JL . Effects of maternal hyperventilation on uterine blood flow and fetal oxygenation and acid–base status. Anesthesiology 1974; 40: 340–7. 4594570 10.1097/00000542-197404000-00007

